# Late-Onset Depression in an Aging World: A Multidimensional Perspective on Risks, Mechanisms, and Treatment

**DOI:** 10.3390/geriatrics11010013

**Published:** 2026-01-26

**Authors:** Antonio Maria D’Onofrio, Gaspare Filippo Ferrajoli, Lodovico Maria Balzoni, Marco Massetti, Andrea Zanzarri, Giuseppe Marano, Marianna Mazza, Alexia Koukopoulos, Georgios D. Kotzalidis, Lorenzo Moccia, Alessio Simonetti, Delfina Janiri, Marco Di Nicola, Gabriele Sani, Giovanni Camardese

**Affiliations:** 1 Department of Neuroscience, Section of Psychiatry, Università Cattolica del Sacro Cuore, 00168 Rome, Italygiorgio.kotzalidis@gmail.com (G.D.K.);; 2 Dipartimento di Salute Mentale, Ospedale di Latina, ASL Latina, 04100 Latina, Italy; 3 Department of Neuroscience, Head-Neck and Chest, Section of Psychiatry, Fondazione Policlinico Universitario Agostino Gemelli IRCCS, 00168 Rome, Italy; 4 Department of Life Science, Health, and Health Professions, Link Campus University, 00165 Rome, Italy; 5 Menninger Department of Psychiatry and Behavioral Sciences, Baylor College of Medicine, Houston, TX 77030, USA

**Keywords:** late-onset depression, vascular depression, neuroinflammation, ageing, late-life depression

## Abstract

**Background:** Late-onset depression (LOD) represents a distinct clinical and biological phenotype emerging in the context of global population ageing. This study aims to synthesize current evidence on the epidemiology, risk factors, mechanistic pathways, and therapeutic approaches of LOD, integrating biological, psychological, and social dimensions. **Methods:** This narrative review synthesizes recent evidence across epidemiology, clinical symptomatology, neurobiology, and treatment. Where conceptually appropriate or empirically overlapping, we incorporate findings from the broader late-life depression (LLD) literature. **Results:** LOD emerges (as a distinct clinical and biological entity in later life) as a clinically and biologically meaningful presentation of depression in later life, representing a minority of depressive cases. It is defined by prominent apathy, psychomotor slowing, and cognitive impairment, and is closely linked to frailty, medical comorbidity, and heightened dementia risk. Pathophysiological mechanisms converge on vascular, inflammatory, oxidative, and neuroplasticity pathways, while psychosocial adversity further shapes onset and course. Treatment prioritizes efficacy and tolerability amid multiple morbidity; SSRIs and SNRIs are first-line, with pro-dopaminergic or dual-action agents addressing anhedonia and apathy, and neuromodulation or augmentation strategies reserved for resistance. Integrative approaches combining pharmacotherapy, psychotherapy, and lifestyle interventions are essential to optimize outcomes in aging populations. **Conclusions:** Late-onset depression (is a distinct, biologically and psychosocially driven disorder) represents a biologically and psychosocially enriched subtype in its own within the spectrum of late-life depression, requiring integrated, personalized care. Addressing neurovascular mechanisms, psychosocial adversity, and prevention through coordinated geriatric and psychiatric strategies may improve outcomes in aging populations.

## 1. Aims of the Review

The present review aims to provide a comprehensive synthesis of current evidence on late-onset depression (LOD)—its epidemiology, clinical features, neurobiological underpinnings, and therapeutic strategies—within the context of global population ageing [1]. While depression in older adults represents a growing public health concern [2,3], LOD, defined as the first major depressive episode (MDE) occurring after age 60–65 [4], it remains underrecognized and insufficiently characterized compared with early-onset forms. (This review seeks to delineate LOD as a distinct clinical and pathophysiological entity, emphasizing its unique interplay with cerebrovascular, neurodegenerative, and psychosocial processes) This review aims to assess the clinical meaning of LOD as a late-life depression subtype within its broader spectrum, characterised by a specific constellation of biological, cognitive, and psychosocial features.

Specifically, the review will: (1) summarize epidemiological trends; (2) examine the clinical, cognitive, and neurobiological distinctions between late-onset and early-onset depression, including the contribution of vascular, inflammatory, and neurotrophic mechanisms; (3) explore the psychosocial and demographic determinants that increase vulnerability in older age; and (4) critically evaluate current treatment approaches, integrating evidence on pharmacological, neuromodulatory, psychotherapeutic, and lifestyle-based interventions tailored to this population.

By consolidating data across neurobiological, psychological, and social dimensions, this review aims to promote a multidimensional understanding of LOD, inform more accurate diagnostic frameworks, and support the development of personalized, multimodal management strategies that optimize both affective and cognitive outcomes in late life.

In some instances, we have also referred to findings from the broader late-life depression (LLD) literature, rather than exclusively from LOD studies, when such evidence was deemed—based on our judgment—conceptually relevant or clinically overlapping with LOD. This approach was adopted to provide a more comprehensive and integrated perspective where distinctions between the two entities are empirically or symptomatologically overtly blurred. Importantly, this conceptualization is intended to be heuristic rather than nosological, and does not imply a categorical separation between LOD and other late-life depressive presentations.

## 2. Methods

This manuscript is a narrative, clinically oriented review aimed at integrating current evidence on late-onset depression across epidemiological, biological, clinical, and therapeutic domains. The selection of thematic areas, level of depth, and overall structure of the review were defined through collegial discussion among all authors, taking into account clinical relevance and the availability and maturity of the existing literature.

Literature searches were conducted across multiple electronic databases, including PubMed/MEDLINE, Scopus, and Web of Science. Search terms were tailored to each section of the review and included combinations of keywords related to late-onset depression, late-life depression, epidemiology, neurobiology, cognitive impairment, and treatment strategies. No restrictions were applied regarding year of publication.

Priority was given, when available, to sources with higher levels of evidence, such as systematic reviews and meta-analyses, followed by large observational studies and randomized controlled trials. Case reports and anecdotal evidence were generally avoided unless considered essential for illustrative or contextual purposes. Study inclusion was based on relevance to the clinical and pathophysiological features of late-onset depression and on overall methodological robustness.

Rather than performing a formal quantitative synthesis, findings were integrated using a narrative approach and organized into thematic sections reflecting key clinical questions. Discrepancies in study interpretation or relevance were resolved by consensus among the authors. The narrative design of the review was chosen to allow a comprehensive and clinically meaningful synthesis of a heterogeneous body of literature.

To enhance transparency, internal consistency, and reporting quality, the preparation of this narrative review was informed by established quality frameworks for narrative reviews. In particular, the SANRA (Scale for the Assessment of Narrative Review Articles) [5] criteria were used as a self-assessment guide during manuscript development and revision, with attention to justification of the article’s importance, clarity of aims, description of the literature search, referencing, scientific reasoning, and appropriate presentation of data. SANRA was used as a reflective framework rather than as a formal scoring tool, in line with the narrative and clinically oriented nature of the review.

## 3. Definition

LOD is defined as a MDE with first onset in later life, most commonly after the age of 60 or 65 years [6,7], although some studies adopt a threshold of 50 years [8]. From a biological standpoint, a lower cut-off (≥50 years) may be advantageous, as several correlates of LOD—including vascular risk factors, alterations in white matter integrity, and dysregulation of gene expression—have been consistently observed in individuals aged 50 years and older, although predominantly in associative studies [9,10]. However, as the global lifespan and population age continue to rise [1], adopting a higher age threshold (60–65 years) may be more appropriate from an epidemiological perspective, better reflecting current demographic patterns. Ultimately, no universally accepted cut-off exists, as both thresholds present distinct advantages and limitations. Accordingly, age of onset should be regarded as a clinically informative dimension rather than a definitive boundary, with late-onset depression reflecting a convergence of aging-related vulnerability and depressive psychopathology.

## 4. Epidemiological Data

Depression is a heterogeneous and multifactorial disorder, whose clinical presentation, course, and response to treatment vary substantially according to the age of onset. While depression affects individuals across all age groups, it also represents a major mental health concern in older adults. It is estimated that approximately 5.7% of people aged 60 years and older meet the criteria for a depressive disorder [11], with prevalence rates increasing progressively with age and peaking among those aged 85 years and above [12]. However, the stringent diagnostic thresholds of the Diagnostic Statistical Manual of Mental Disorders (DSM-5) likely underestimate the true burden of depressive morbidity in late life, as many older adults presenting clinically relevant depressive symptoms fail to meet full diagnostic criteria for major depressive disorder (MDD) [13]. In a recent population-based study of 1304 community-dwelling older adults, only 4% received a formal diagnosis of depression, whereas 27% reported depressive symptoms [14]. This discrepancy highlights the substantial gap between diagnosed depressive disorders and subthreshold depressive symptomatology, underscoring the need to consider both syndromic and subclinical manifestations when estimating the true burden of depression in ageing populations.

The probability of experiencing a first MDE varies markedly across the lifespan. Epidemiological data indicate that the highest risk period for depression onset extends from adolescence through mid-adulthood, with a mean age of onset between 25 and 29 years [15,16]. Approximately 40% of individuals experience their first depressive episode before age 20, while the majority of cases occur between 18 and 44 years [15,17,18]. The incidence of new depressive episodes peaks during the third decade of life, followed by a secondary, smaller peak between ages 45 and 59. Analyses using Life Table methods have shown that the risk of a first episode increases sharply during adolescence, peaks in early adulthood, and gradually declines in later life [19]. Additional evidence indicates that the cumulative incidence of MDD may reach up to 51% by early adulthood, confirming the strong age dependency of depression onset [20].

Within this broader epidemiological framework, LOD represents a distinct minority of cases. Its relative rarity emphasizes its likely pathophysiological specificity, as LOD departs from the typical age distribution of depression onset and is presumed to arise from neurobiological and systemic processes linked to ageing, including cerebrovascular and neurodegenerative mechanisms [21,22]. Importantly, however, the epidemiological characterization of LOD is intrinsically dependent on how late onset is operationally defined. Indeed, estimated prevalence and incidence vary substantially according to the age cut-offs adopted across studies, which range from 50 to 65 years. Lower thresholds (≥50 years) tend to inflate prevalence estimates by encompassing a broader and potentially more heterogeneous group, whereas higher cut-offs (60–65 years) yield smaller but epidemiologically more specific samples that better align with contemporary ageing demographics. This definitional variability complicates direct cross-study comparisons and highlights the need to interpret LOD epidemiological data in close relation to the operational criteria applied.

## 5. Late-Onset Depression (LOD) Versus Early-Onset Depression (EOD) and Late-Life Depression (LLD)

Depression in later life is a clinical entity with diverse etiological pathways and prognostic implications. Among the most debated distinctions in geriatric psychiatry is the differentiation between LOD and EOD, particularly within the broader framework of LLD. While LLD encompasses both recurrent or persistent depressive episodes originating in early adulthood and those with onset in old age, a growing body of literature suggests these two presentations are clinically, cognitively, and biologically distinct [23,24]. At the same time, symptom profiles show substantial overlap, and distinctions are often quantitative rather than qualitative. A systematic review and meta-analysis by Hegeman et al. [25] demonstrated that, compared with younger adults, older individuals with major depression more frequently exhibit agitation and general or gastrointestinal somatic symptoms, whereas feelings of guilt and loss of sexual interest are less prominent. However, no statistically significant differences were observed for several core depressive features, including late insomnia, somatic anxiety, and suicidality, underscoring the considerable symptomatic overlap between age-of-onset groups. Overall, these findings suggest that depression in older age may be characterized by a relative shift toward somatic symptom expression rather than by a distinct symptom constellation.

LLD often presents with a unique set of symptoms that do not always align with the diagnostic criteria for depressive disorders as outlined in the DSM-5 [11]. Despite this, these symptoms carry significant clinical weight, as they are strongly associated with negative health outcomes and a diminished quality of life. The frequent under-recognition of LLD means that many individuals do not receive the necessary care and treatment. It is crucial to note that LLD is not a temporary or self-resolving condition; it typically requires psychopharmacological or psychological interventions [26]. LLD is categorized based on the age at which depressive symptoms first appear. When depression begins earlier in life (during youth or middle age) and persists into older adulthood, it is referred to as Early-Onset Depression (EOD) [22]. Conversely, LOD is diagnosed when depressive symptoms emerge for the first time in later life, with no prior history of major depressive episodes [27]. While there is no universally agreed-upon age cutoff to distinguish EOD from LOD, thresholds of 55 or 60 years old are commonly used. Both subtypes are believed to contribute roughly equally to the overall prevalence of LLD [28]. These findings support the view that differences between LOD, EOD, and LLD are largely dimensional, reflecting shifts in symptom expression, cognitive involvement, and biological burden, rather than discrete diagnostic categories.

## 6. “Primary” LOD vs. “Secondary” LOD

Moreover, LOD itself can be further subclassified into “primary” forms, presumed to be idiopathic, and “secondary” forms, which are associated with identifiable neurological, systemic medical conditions or pharmacologic triggers [29,30]. Primary LOD is typically diagnosed when no clear somatic or iatrogenic cause for the depressive episode can be identified. These patients often present with symptoms such as apathy, anhedonia, executive dysfunction, and psychomotor slowing, without a prior psychiatric history [23]. Neuroimaging studies have consistently shown that individuals with primary LOD exhibit increased burden of white matter hyperintensities (WMH), particularly in fronto-striatal and limbic circuits, supporting the “vascular depression” hypothesis [31]. Microstructural disruptions in these networks are thought to impair top-down regulation of mood and affect, leading to depressive symptoms resistant to conventional antidepressants, especially those that target serotonergic pathways alone [32].

The distinction between primary and secondary LOD is not merely academic but has significant clinical implications. For example, in elderly individuals presenting with first-onset depression accompanied by apathy, executive dysfunction, and subtle neurologic signs, a detailed work-up including neuroimaging, metabolic screening, and review of pharmacological history is warranted to exclude a secondary form [31]. Similarly, LOD in patients with recent-onset parkinsonism or gait disturbances may herald the development of neurodegenerative disorders such as Parkinson’s disease or progressive supranuclear palsy, necessitating early neurological referral [33].

Growing attention has been paid to the potential neurodegenerative underpinnings of primary LOD. Longitudinal studies have demonstrated that patients with first-onset depression after age 65 have a significantly elevated risk of developing Alzheimer’s disease (AD) or other dementias, even after accounting for baseline cognitive function and vascular risk factors [34,35]. This has led to the hypothesis that in a subset of patients, primary LOD may represent a prodromal phase of neurodegenerative disease. Supporting this are findings showing that depression was significantly associated with the presence of subcortical Lewy bodies and with neuronal loss in the hippocampus as well as in several subcortical structures, including the nucleus basalis, substantia nigra, and raphe nucleus [36]. Thus, primary LOD may serve as an early clinical biomarker for the development of AD or frontotemporal dementia (FTD). Inflammatory and immune pathways have also been implicated in primary LOD. Several studies have documented elevated peripheral levels of pro-inflammatory cytokines, such as interleukin-6 (IL-6), tumor necrosis factor-alpha (TNF-α), and C-reactive protein (CRP), in older adults with depression [37,38]. These molecules may access the central nervous system (CNS) through a compromised blood–brain barrier (BBB), triggering microglial activation and synaptic remodeling. The result is a low-grade neuroinflammatory environment that may disrupt neural plasticity, particularly in prefrontal and limbic areas [39,40,41,42,43,44]. These mechanisms offer a plausible link between depression, vascular risk factors (e.g., hypertension, diabetes), and neurodegeneration. In contrast, secondary LOD is diagnosed when the depressive syndrome is temporally and causally linked to a medical illness or pharmacological treatment. Neurological conditions such as Parkinson’s disease, stroke, multiple sclerosis, epilepsy, and traumatic brain injury are among the most commonly associated with secondary LOD [29,45,46]. In such cases, depression may derive from direct injury to emotion-regulating networks (e.g., basal ganglia, anterior cingulate cortex), inflammatory processes, or neurochemical alterations involving dopaminergic, serotonergic, or noradrenergic pathways. For instance, post-stroke depression is well recognized and has been associated with lesions in the left frontal lobe and basal ganglia, highlighting the role of lesion location in mood regulation [47].

Similarly, endocrine and metabolic disorders, such as hypothyroidism, Cushing’s disease, diabetes mellitus, and systemic lupus erythematosus, can precipitate depressive syndromes in later life. These conditions may cause or exacerbate LOD through mechanisms including HPA axis dysregulation, cytokine release, and glucocorticoid receptor resistance [15,29,48,49,50,51]. Additionally, many commonly prescribed medications in the elderly population have been implicated in the onset of secondary LOD. These include corticosteroids, interferon-alpha, beta-blockers, certain anticonvulsants (e.g., levetiracetam), and dopaminergic agents used in Parkinson’s disease [52]. The time course of symptom emergence following initiation or dosage escalation of these agents can provide important diagnostic clues.

Secondary LOD may present with more prominent somatic and cognitive symptoms, and may lack the typical affective features of primary depressive episodes [53]. Importantly, secondary LOD often shows limited or no response to standard antidepressants unless the underlying cause is addressed. This makes diagnostic accuracy essential, as misclassification may lead to ineffective treatment and prolonged functional impairment. In some cases, discontinuation of the offending medication or treatment of the medical condition can lead to full resolution of depressive symptoms.

Despite these distinctions, primary and secondary LOD can co-occur or transition over time. For example, a patient with long-standing type 2 diabetes may initially develop LOD due to vascular and metabolic factors, but later evolve a neurodegenerative process that amplifies depressive symptoms. Alternatively, chronic use of corticosteroids in an elderly patient may induce depressive symptoms that unmask a latent vulnerability to mood dysregulation [54]. Thus, a dynamic, biopsychosocial model is required to understand the evolution of LOD in individual patients.

**Figure 1:**
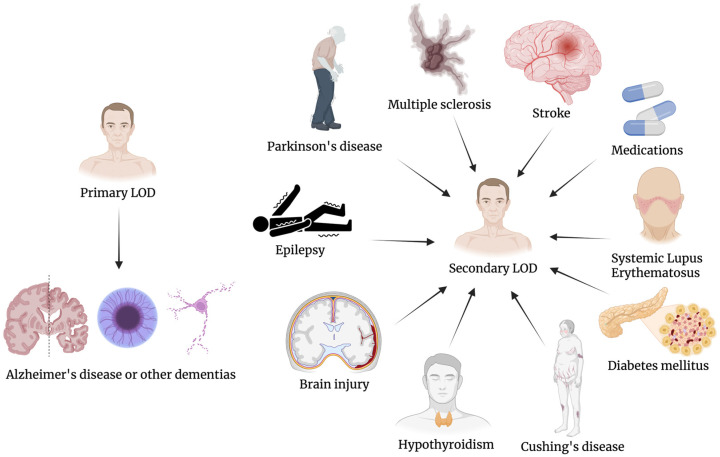
Primary and Secondary Late-Onset Depression (LOD). Primary LOD refers to idiopathic depressive syndromes emerging in later life, typically characterized by apathy, anhedonia, executive dysfunction, and psychomotor slowing in the absence of overt neurological or systemic disease. In a subset of patients, primary LOD may represent a prodromal phase of neurodegenerative disorders such as Alzheimer’s disease or other dementias. Conversely, secondary LOD arises from identifiable neurological, systemic, or pharmacologic causes that converge on mood-regulating circuits. Neurological conditions (e.g., Parkinson’s disease, stroke, multiple sclerosis, epilepsy, traumatic brain injury) and systemic–endocrine disorders (e.g., diabetes mellitus, hypothyroidism, Cushing’s disease, systemic lupus erythematosus) can induce depression via neuroinflammation, hypothalamic–pituitary–adrenal (HPA) axis dysregulation, and monoaminergic imbalance. Figure created in BioRender.com.

We summarized in Figure 1 the conceptual distinction between primary and secondary forms of LOD.

## 7. Psychosocial and Demographic Risk Factors

LOD results from a complex interaction of biological, psychological, and social factors that differentiate it from early-onset depression. Importantly, psychosocial factors should not be viewed solely as downstream triggers of depressive episodes, but as dynamically interacting with biological vulnerability through bidirectional mechanisms, including stress-related neuroendocrine, inflammatory, and neuroplastic pathways. Beyond neurodegenerative and cerebrovascular mechanisms, psychosocial and demographic factors—such as advanced age, female gender, low socioeconomic status, limited social support, medical comorbidities, and stressful life events—play a crucial role in its onset and progression [55,56]. Unlike early-onset depression, where genetic predisposition and family history are more prominent, LOD often emerges in the context of environmental vulnerability, highlighting the importance of non-biological domains in its pathogenesis. A range of non-biological factors has been implicated in the risk for LOD.

Demographic risk factors are among the most well-established predictors of LOD. Age itself is a key risk factor, not only due to cumulative physiological decline but also because of increased exposure to loss and social disconnection. Epidemiological studies have consistently shown a higher prevalence of depressive symptoms among the oldest old (i.e., individuals aged ≥85), suggesting a dose–response effect of aging on emotional well-being [57,58].

The association between advancing age and depression follows an inverted J-shaped curve [59]. Individuals aged between 70 and 80 years exhibit a higher likelihood of experiencing depressive symptoms compared to those under 70, while this risk does not appear to increase further in adults over 80 [60].

Gender is another important determinant: older women are more likely than men to experience LOD, a disparity attributed to hormonal fluctuations, longer life expectancy, greater likelihood of widowhood, and higher rates of caregiving burden [61,62]. Data from a nationwide U.S. survey also revealed a significantly higher prevalence of depressive symptoms in women (26.9%) compared to men (19.9%) [63].

Social factors, such as marital status and living arrangements, also contribute to LOD risk. A large cross-sectional study in Japan highlighted a connection between depressive symptoms and experiences like separation or divorce, as well as financial debt [64]. These factors can exacerbate psychological distress due to social isolation or economic hardship. Similarly, research involving older adults in rural China found that high scores on the CESD-10 depression scale were linked to low personal income, the use of polluting cooking fuels, a lack of seated toilets, and an absence of bathing facilities [65]. Further evidence from Japan suggests that socioeconomic conditions may have a long-term impact on the risk of developing depression, with retirement identified as another contributing factor to its onset [66]. Moreover, individuals with lower education and income levels face higher risk of depression in old age, possibly due to lifelong accumulation of stress, reduced access to healthcare, and poorer health literacy [67].

Cultural and environmental factors play a critical role in shaping the risk for LOD. In many settings, the stigma associated with mental illness may discourage older adults from seeking help [68], leading to underdiagnosis and delayed treatment. Furthermore, disparities in access to mental health services, particularly in rural or socio economically disadvantaged areas, can exacerbate existing vulnerabilities [69]. Environmental stressors such as displacement, limited mobility, or substandard living conditions may further contribute to psychological distress in later life, especially when combined with social isolation or bereavement [70,71]. These contextual determinants highlight the importance of considering broader sociocultural dynamics in both the prevention and management of LOD.

Psychosocial factors such as adverse life events, reduced social networks, a sense of being alone, and lower financial standing are known to be associated with LLD [71,72]. Even prior to the COVID-19 pandemic, feelings of loneliness were acknowledged as a major psychological stressor among older adults. A large-scale, long-term study of older individuals revealed that 43% reported experiencing loneliness [73]. Another study in an elderly Finnish population group showed that prolonged patterns of depression were influenced by persistent feelings of being alone [74].

Social isolation and loneliness represent potent psychosocial triggers of LOD. Unlike mere physical solitude, loneliness reflects the subjective perception of social disconnection and is strongly associated with both incident and persistent depressive symptoms [75]. Social isolation and a lack of access to supportive relationships have been connected to moderate and severe symptoms of depression in older individuals [76]. Social isolation is highly prevalent in older adults, particularly those who are widowed, live alone, or reside in long-term care facilities [77]. A systematic review by Santini et al. (2015) found that perceived loneliness increases the risk of subsequent depressive symptoms by over 40%, independent of baseline health status [78].

Research by Chi and Chou established a strong link between the degree of social support and depressive symptoms across several measures. These included the size of a person’s social network, the composition of that network, how often they interact, how satisfied they are with the support they receive, the availability of emotional and practical help, and opportunities to assist others [79]. Furthermore, a study by Lee et al. (2021) showed that higher loneliness scores at baseline were associated with greater depressive symptom severity over a 12-year follow-up among adults aged 50 years and older [80].

Bereavement and stressful life events are also salient contributors to the pathogenesis of LOD. Loss of a spouse, close friend, or adult child is common in late life and represents a significant psychological insult, particularly when compounded by poor coping resources or previous trauma [81]. While grief is a normative process, it may transition to pathological depression in vulnerable individuals. Studies indicate that 15–30% of bereaved elders develop clinically significant depressive symptoms within the first year of loss [82]. Furthermore, older adults are not immune to other forms of psychosocial stress, including financial strain, physical disability, elder abuse, and role transitions (e.g., retirement), all of which may trigger or sustain depressive episodes [61].

Complicated grief disorder has symptoms that overlap with LOD and can occur at the same time; both conditions have been linked to higher rates of illness and death. The period of highest relative mortality risk following the death of a spouse has been found to be between 7 and 12 months after the loss [83].

Bereavement is a psychosocial challenge known to increase the risk of suicide. Therefore, evaluating suicide risk is a critical part of the clinical assessment for individuals with LOD, especially after a significant loss. Identifying a lack of social support, monitoring for suicidal ideation or behaviors, and providing specific follow-up for those at increased risk are key prevention strategies. For instance, the Prevention of Suicide in Primary Care Elderly: Collaborative Trial (PROSPECT) was an innovative initiative in primary care settings designed to reduce suicide risk and prevent negative health outcomes among older adults [84].

Elderly individuals generally maintain a limited number of emotionally meaningful connections while reducing their participation in wider social circles [85]. Conversely, possessing more social ties and feeling well supported have been identified as protective factors against LOD, and are associated with better results from depression therapy [86]. These beneficial effects are believed to function through multiple mechanisms, such as providing emotional comfort, practical assistance, or increasing chances for enjoyable social engagement [87]. Recent research has focused on the idea of loneliness, which is the personal feeling of being socially isolated, regardless of the actual number of social interactions a person has. Loneliness has been shown to have a two-way relationship with several negative health outcomes, including symptoms of depression, declining physical health, reduced cognitive and functional abilities, and a higher risk of death [88]. It has also been suggested that loneliness could be an early neurobehavioral sign of preclinical Alzheimer’s disease, given its connections to a greater chance of developing dementia and to increased levels of amyloid and tau protein buildup in the brain [89].

Resilience factors also modulate risk for LOD. Personality traits such as neuroticism, low conscientiousness, and low extraversion are linked to poorer adaptation to age-related stressors [90]. Moreover, the presence of strong social support, meaning-making capacity, religious faith, and a sense of coherence can buffer against the development of depression even in the context of adverse life events [91]. Psychosocial interventions aimed at enhancing resilience, coping strategies, and perceived control have shown promising results in preventing or mitigating depressive symptoms in at-risk elderly populations [92].

Finally, life-course exposures play a cumulative role in shaping the vulnerability to LOD. Early adversity, including childhood trauma, poverty, and neglect, has been linked to altered stress reactivity, epigenetic modifications, and lifelong mental health risk [93]. While these factors are more classically associated with EOD, their influence can persist or resurface in late life, particularly when combined with new stressors or medical decline.

In summary, late-onset depression is not solely a neurobiological or neurodegenerative disorder, but also deeply rooted in psychosocial and demographic vulnerabilities. Across the life course, psychosocial adversity may both contribute to and result from biological susceptibility, reinforcing depressive risk through reciprocal pathways involving stress physiology, immune activation, cerebrovascular burden, and neurodegenerative processes. Social isolation, medical comorbidities, stressful life events, and socioeconomic disadvantage all contribute to the onset and persistence of depression in older adults. Comprehensive assessment and intervention strategies that address both medical and psychosocial domains are essential to effectively manage and prevent LOD. Public health policies aimed at reducing loneliness, enhancing community support, and improving access to care for older adults may have a substantial impact on reducing the burden of this complex and often underdiagnosed condition.

Table 1 provides an overview of these demographic and psychosocial contributors, which may interact with neurobiological mechanisms to trigger depressive episodes in older adults.

## 8. Biological Mechanisms

While EOD is often linked to genetic predisposition and early neurodevelopmental factors, LOD appears to emerge from a confluence of age-related biological changes, including cerebrovascular decline, chronic low-grade inflammation, neurodegeneration, and impaired neuroplasticity. This distinction is intended to clarify biological prioritization rather than to establish exclusive causal hierarchies.

Among the most studied mechanisms are vascular dysfunction, inflammatory processes, accelerated brain aging, and altered levels and signaling of brain-derived neurotrophic factor (BDNF). Understanding these biological underpinnings is crucial for developing effective prevention and treatment strategies tailored to the aging population.

Importantly, these mechanisms should not be interpreted as equivalent causal drivers. Rather, some processes appear more closely linked to aging-related vulnerability (e.g., cerebrovascular pathology and immunosenescence), whereas others may represent downstream mediators or correlates that modulate symptom expression, illness course, or treatment response. Within this framework, aging-related biological vulnerability is primarily shaped by upstream processes, including cerebrovascular pathology and immunosenescence, which progressively alter brain structure, perfusion, and immune–brain communication, thereby increasing susceptibility to depressive syndromes in later life. In contrast, downstream mechanisms—such as chronic inflammation, oxidative stress, HPA-axis dysregulation, mitochondrial dysfunction, and alterations in BDNF signaling—are best conceptualized as interacting pathways that both reflect and amplify this underlying vulnerability. These biological processes are discussed individually in the following sections, in order to clarify their specific contributions and points of interaction within the pathophysiology of late-onset depression.

### 8.1. Vascular Disease and the Vascular Depression Hypothesis

The onset or worsening of depressive symptoms after the development of vascular disease, the prevalence of cerebrovascular risk factors in people with depression, and the specific types of cognitive deficits linked to the location and extent of cerebral damage are all key pieces of evidence. Additionally, the limited effectiveness of traditional antidepressant medications in these cases further strengthens this theory [94]. A leading explanatory model for LOD is the vascular depression hypothesis, proposed by Alexopoulos et al. [95]. This model suggests that cerebrovascular disease, particularly small vessel disease, contributes to the pathophysiology of depression in older adults [95]. While some neuropathological research has not substantiated the vascular hypothesis [96,97], other studies indicate that the presence of cerebral small vessel disease is associated with a greater risk of depressive symptoms in older adults, regardless of their cognitive status [98,99,100,101].

Neuroimaging studies have shown decreased blood flow in areas such as the precuneus, cuneus, and fronto-cingulate-striatal circuits, as well as in temporal, occipital, and parietal lobes. Researchers have also identified changes in brain activity during a resting state that are characteristic of depressive conditions, along with heightened activity in the limbic system [102]. Taylor and colleagues have consistently shown that individuals with LOD exhibit higher volumes of white matter hyperintensities (WMHs), particularly in frontal-subcortical circuits that regulate mood and executive function [53].

These conclusions are further corroborated by a significant postmortem study of elderly individuals who had a history of depression but no signs of dementia [103]. These microscopic brain changes disrupt cortico subcortical circuits, which are crucial pathways for controlling emotions and behavior. This disruption provides a solid basis for a link between CSVD and depressive symptoms among the aging population [104,105].

Moreover, the presence of increased circulating biomarkers related to endothelial dysfunction and reduced flow mediated vasodilation offers further physiological proof for the vascular origins of some depressive disorders [102]. In addition, reduced cerebral perfusion and chronic hypoxia may exacerbate neurodegenerative processes. The vascular hypothesis is further supported by findings that LOD patients often exhibit executive dysfunction and are less likely to respond to standard antidepressant treatments compared to those with early-onset depression. However, the hypothesis is not without its limitations. The variable course of depressive symptoms over time and the inconsistent link between the size of a stroke and the severity of depression cast doubt on its explanatory completeness [94]. Furthermore, not everyone who suffers from an ischemic brain injury develops depression, which suggests other contributing factors must be at play. Epidemiological studies have investigated the relationship between depression, dementia, and cardiovascular diseases, including heart failure. However, the influence of depression on patient outcomes and the role of heart failure as a possible risk factor for developing dementia are topics that still need more thorough investigation [106].

### 8.2. Chronic Inflammation and Neuroimmune Dysregulation

Disruptions in immune function, whether resulting from aging or underlying disease, appear to be a key factor [94]. The wide range of age-related changes that affect immune responsiveness is collectively referred to as “immunosenescence” [107]. Multiple cross-sectional studies have found higher levels of proinflammatory substances in older adults [108]. Furthermore, weakened or suppressed immune responses are thought to contribute to various problems associated with immune aging. In particular, chronic proinflammatory activity may disrupt communication between the peripheral immune system and the central nervous system, diminish normal anti-inflammatory defenses, and foster a persistent neuroinflammatory environment marked by increased production of proinflammatory cytokines and reactive oxygen and nitrogen species, such as interleukin-6 (IL-6), tumor necrosis factor-alpha (TNF-α), and C-reactive protein (CRP) [109]. These inflammatory mediators can penetrate the blood–brain barrier, particularly when it becomes more permeable with age, and interfere with neurotransmission, synaptic plasticity, and neurogenesis [110].

Elevated levels of inflammatory markers in individuals with LOD have been described [111]. Chronic inflammation may impair the serotonergic system by shifting tryptophan metabolism away from serotonin production toward the kynurenine pathway, resulting in the accumulation of neurotoxic metabolites such as 3-hydroxykynurenine and quinolinic acid [112]. These compounds can damage neurons, activate microglia, and contribute to depressive symptomatology. Inflammatory cytokines also play a role in hippocampal atrophy and cortical thinning, neuroanatomical changes frequently observed in late-life depression. Persistent neuroinflammation may therefore act as a bridge between depressive syndromes and neurodegenerative diseases like Alzheimer’s, with which LOD shares several pathophysiological features [113].

### 8.3. Brain Aging and Neuronal Vulnerability

Aging itself profoundly alters brain structure and function. Normal aging is characterized by reductions in total brain volume, particularly in the prefrontal cortex and hippocampus, and by declines in synaptic density, dendritic arborization, and neurogenesis [114]. These changes may compromise the brain’s ability to adapt to stress and environmental demands, thereby increasing susceptibility to depression. A large body of the literature has documented a decrease in cortical thickness across various brain areas. These regions include the prefrontal and orbitofrontal cortex, both parts of the cingulate cortex (anterior and posterior), as well as sections of the temporal and parietal lobes, hippocampus, amygdala, striatum, thalamus, and insula. These structural changes are also correlated with cognitive impairment, a key feature of LOD [105]. Reduced hippocampal volume has been frequently associated with later onset of depressive episodes, longer illness duration [115], and a higher chance of relapse [116], which could explain the observed link between a smaller hippocampus and LLD [117]. However, a prospective longitudinal study did not confirm this relationship [118].

Mitochondrial dysfunction and oxidative stress are also hallmarks of brain aging. Neurons, especially in regions involved in emotion regulation, become more vulnerable to metabolic insults, potentially amplifying the effects of other risk factors such as vascular damage or inflammation [119]. Studies consistently support the hypothesis that depression in later life is linked to irregularities in oxidative stress markers and mitochondrial dysfunction [120]. The relationship between this oxidative imbalance, impaired mitochondrial function, and depression might be facilitated by the activation of sterile inflammatory pathways and damage associated molecular patterns (DAMPs) [121]. The buildup of cellular harm from oxidative injury and mitochondrial damage leads to the release of DAMPs [122]. These molecular signals, in turn, intensify immune activation. Notably, mitochondrial DNA not only initiates a type I interferon response but also activates Toll-like receptor 9 and the NLRP3 inflammasome, thus promoting neuroinflammation [123]. Furthermore, an overproduction of reactive oxygen species (ROS), when coupled with elevated oxidative stress and weakened antioxidant systems, can negatively affect neuronal health and synaptic signaling [124]. A heightened oxidative burden may also contribute to further mitochondrial degradation, increased programmed cell death, and the amplification of inflammatory signaling cascades [125], all of which have been connected to behaviors seen in depressive states.

Additionally, age-related dysregulation of the HPA axis may lead to elevated and prolonged cortisol levels, which have been linked to hippocampal shrinkage and impaired feedback inhibition of the stress response [126]. These biological changes help explain why LOD is often associated with poorer treatment outcomes, greater cognitive impairment, and increased risk of conversion to dementia. Importantly, they also highlight the need for interventions that go beyond symptom reduction and aim to preserve or restore neurocognitive resilience.

### 8.4. Alterations in BDNF and Neuroplasticity

Brain-derived neurotrophic factor (BDNF) is highly concentrated in the central nervous system, especially in the hippocampus, where it is essential for neuroplasticity [127,128]. BDNF plays a crucial role in maintaining the growth, specialization, and maintenance of neurons throughout life [129]. It is a key regulator of synaptic plasticity in brain circuits associated with many psychological processes, including mood regulation, and with various psychiatric disorders, including depressive disorders [130,131]. Decreased levels of BDNF have been consistently reported in patients with depression, including those with depression in later life [132]. In the aging brain, BDNF expression is downregulated by chronic stress, inflammation, and reduced neuronal activity, factors all commonly present in late-life depressive states [133]. Furthermore, increased BDNF expression can help counteract the deficits in structural and synaptic plasticity caused by stress, thereby supporting cognitive resilience and protecting against depressive symptoms [134,135].

BDNF is particularly active in the hippocampus and prefrontal cortex, brain regions essential for memory, learning, and mood regulation. Its expression is modulated by activity-dependent pathways and is negatively affected by pro-inflammatory cytokines and glucocorticoids. Preclinical studies have shown that BDNF infusion can reverse depressive-like behaviors in animal models, while BDNF depletion leads to affective and cognitive symptoms [136].

Alterations in BDNF signaling may also contribute to the reduced efficacy of antidepressant treatments in older adults. Many antidepressants, including SSRIs and SNRIs, are thought to exert part of their therapeutic effect by enhancing BDNF expression. Moreover, non-pharmacological interventions such as exercise, cognitive training, and neuromodulation techniques like transcranial magnetic stimulation have been shown to increase BDNF levels [137]. Genetic polymorphisms, such as the BDNF Val66Met variant, may further modulate individual responses to both environmental stressors and therapeutic interventions, highlighting the importance of personalized approaches in the management of LOD [138].

Individuals affected by depression and dementia consistently show reduced BDNF concentrations, while antidepressant medications have been found to increase these levels [139,140]. Both genetic variations and blood levels of BDNF have been linked to affective symptoms like depression and anxiety, as well as personality traits such as neuroticism and the modulation of serotonergic signaling pathways [141,142,143]. The addition of lithium, a common treatment for treatment resistant depression, has also been shown to boost BDNF expression [140]. A frequent symptom among people with depression is sleep disturbance, particularly insomnia [144]. In this context, Giese et al. (2014) identified a link between BDNF levels and sleep quality, reporting that individuals with insomnia had significantly lower concentrations of the neurotrophins [145].

The pathology of LLD appears to be rooted in the cumulative effects of aging, which orchestrate a cascade of biological changes that compromise brain function. A central theme is the critical role of vascular disease, as impaired cerebral blood flow leads to microvascular damage and subsequent structural changes that disrupt key brain circuits. This process is exacerbated by chronic, low-grade inflammation, which can negatively impact neuronal health and function. Furthermore, the overall process of brain aging contributes to a reduction in neuronal plasticity and resilience. Crucially, these factors converge to alter key signaling pathways, including a decline in BDNF, a protein essential for neuronal growth and survival.

Taken together, these mechanisms point to LOD as a distinct clinical entity driven by an intricate interplay of vascular, inflammatory, and neurotrophic factors. This integrated perspective necessitates a reevaluation of therapeutic approaches, suggesting that effective interventions should aim to target these underlying biological vulnerabilities rather than relying solely on treatments that address neurotransmitter imbalances.

**Figure 2:**
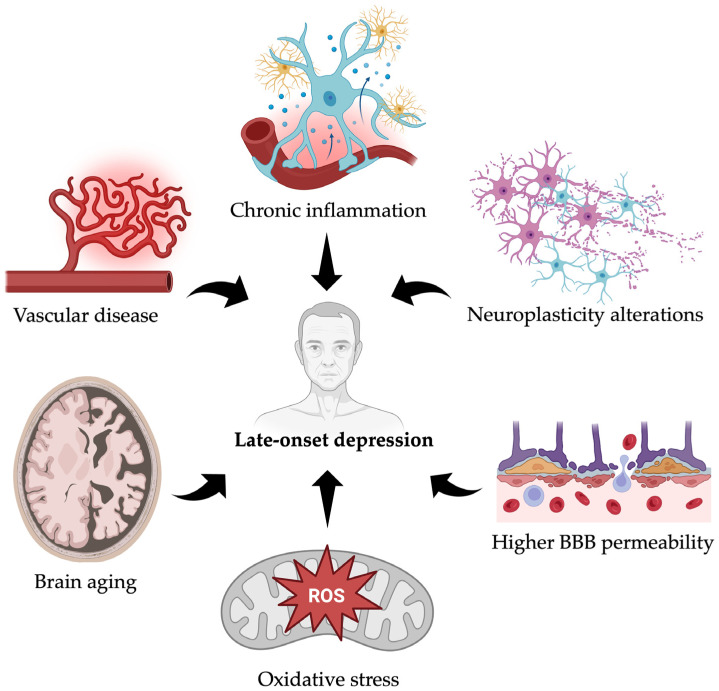
Biological mechanisms implicated in Late-Onset Depression (LOD). LOD is underpinned by a multifactorial pathophysiology involving the convergence of vascular decline, chronic low-grade inflammation, oxidative stress, neuroplasticity alterations, increased blood–brain barrier (BBB) permeability, and accelerated brain ageing. These interrelated processes disrupt neuronal integrity and neurovascular coupling, contributing to affective and cognitive symptoms characteristic of LOD. Figure created in BioRender.com.

In Figure 2, we summarize the main biological mechanisms implicated in the pathophysiology of Late-Onset Depression, highlighting the interplay among vascular disease, chronic inflammation, oxidative stress, increased blood–brain barrier permeability, neuroplasticity alterations, and brain ageing.

## 9. Clinical Presentation and Diagnosis

When depression first appears in later life, the clinical picture often revolves around low drive and energy, apathy, psychomotor slowing, irritability, and a dense layer of somatic/pain complaints—the classic “masked” presentation described in geriatric psychiatry [146]. Anhedonia and lack of motivation, rather than overt sadness, tend to anchor the late-onset signal, which helps explain under-recognition in primary care [147]. Cognitive complaints are common from the outset. In treated outpatients older than 60, LOD is linked to poorer memory and more dyskinesias than earlier-onset trajectories [6]. At the population level, LOD also carries a temporal association with subsequent dementia [148]. Because cognitive symptoms are so salient in this group, it is sensible to establish a baseline cognitive profile for differential diagnosis and longitudinal follow-up [148,149,150] helping to distinguish depression-related cognitive dysfunction—which may improve with symptom remission—from cognitive decline driven by prodromal neurodegenerative processes. Beyond mood and cognition, several studies have examined odor identification as a pragmatic adjunct in LOD. While not diagnostic, impairment on these tasks has been discussed as relevant to dementia risk in depressed elders [151,152]; however, current evidence remains preliminary, heterogeneous, and insufficient to support their routine clinical use. DSM-5 criteria remain the syndromic anchor, but assessment in geriatrics should work around multiple morbidity, polypharmacy, sleep and pain syndromes, and evolving cognitive change [153]. For screening and monitoring, the Geriatric Depression Scale is practical, since it uses a yes/no format and de-emphasizes somatic and vegetative items—features intended to reduce confounding from medical illness in older patients [147,153]. In specialty settings, Hamilton Depression Rating Scale (HAM-D) and Montgomery–Åsberg Depression Rating Scale (MADRS) remain the main tools for rating severity and response, having been used large European real-world characterization studies of LOD [31]. The short list to exclude—before concluding primary LOD—includes prodromal neurodegeneration, vascular contributions, medication effects, and sleep or pain disorders [154]. Two recurrent objective signals help triage, i.e., meta-analytic magnetic resonance imaging (MRI) work showing greater white-matter hyperintensity burden in LOD, consistent with a vascular pathway [155]; and PET-oriented syntheses, describing amyloid/tau positivity in a subset of geriatric depression cases, consistent with prodromal neurodegeneration in some of them [152]. Medication and comorbidity reviews are mandatory at every step [153,156].

## 10. Clinical and Functional Consequences

Over long horizons, even subsyndromal depression in late life carries weight: in a 15-year cohort, both major and subthreshold depression accelerated multiple morbidity, with a “cognitive” symptom phenotype tracking the steepest deterioration [157]. Classic geriatric reviews converge on the same point—functional losses and medical vulnerability amplify the impact of modest depressive burden in older adults [55]. In remitted LLD, higher perceived stress, disability in instrumental activities of daily living and lower instrumental social support predicted recurrence; a history of >3 depressive episodes also predicted recurrence in a secondary model [158]. Vascular conditions, such as hypertension, more often affect LOD patients, contributing to greater overall medical vulnerability; depression in late life is also associated with increased mortality in the broader geriatric literature, although evidence is inconsistent [8,55]. Reviews further point to microvascular dysfunction as an early, potentially targetable mechanism co-occurring with cognitive inefficiency and apathy phenotypes [104]. Beyond global quality-of-life effects, specific symptom domains matter: apathy is frequent and disabling in LLD and tracks with functional outcomes over treatment courses [159]. Also, six-month follow-up in clinically treated late-onset cases found cognitive impairment was frequent at baseline and often persisted despite mood improvement [160]. Appetite and weight-loss symptom factors have also been associated with broader neurocognitive deficits in late-life depression [161]. At a systems level, depression in later life shows a robust longitudinal association with frailty, with reciprocal links to disability and cognitive decline [162]. Clinically, LOD is linked to poorer memory and more dyskinesias than earlier-onset illness presenting in later life [6]; in longitudinal work, LOD declined faster in verbal skills and delayed memory than both controls and early-onset late-life depression [150]. In specialty care, patients with LOD nevertheless showed lower current suicidal risk than those with earlier onset [8]. Review data also indicate that LOD/LLD tracks with increased risk of subsequent dementia, supporting cognitive monitoring after new late-life episodes [148,150]. First-episode adult data suggest later onsets are more tied to environmental precipitants than the trait vulnerabilities seen in early onsets, which is useful for shaping psychosocial planning [163].

## 11. Therapeutic Strategies

### 11.1. Pharmacological Interventions

The management of LOD requires consideration of the unique biological and clinical features that characterize this subtype. Pharmacological treatment is often complicated by age-related physiological changes, medical comorbidities, and polypharmacy, which make drug selection less straightforward than in younger patients, demanding a careful balance between efficacy, tolerability, and potential drug–drug interactions [164]. In this section, we focus primarily on antidepressant agents and therapeutic strategies that specifically target the core clinical features of LOD. Other pharmacological classes—including mood stabilizers and antipsychotics—are not discussed in detail, although they may be employed in selected clinical contexts (e.g., treatment-resistant or psychotic depression). This focused approach aims to highlight treatments most directly relevant to the pathophysiological substrates of LOD.

#### 11.1.1. SSRI and SNRI

SSRIs and SNRIs are generally considered first-line options compared with tricyclic antidepressants, which carry a non-negligible risk of anticholinergic and cardiotoxic effects in older adults [165]. Among SSRIs, citalopram, escitalopram, and sertraline are the most commonly used in the elderly: all act by enhancing serotonergic transmission and modulating the fronto-limbic circuits involved in mood regulation. SNRIs, such as venlafaxine and duloxetine, add a noradrenergic reinforcement to the serotonergic mechanism, providing greater impact on apathy, psychomotor retardation, and somatic symptoms, particularly chronic pain [166].

The efficacy of these agents in older adults has been widely investigated. An interesting finding comes from Nelson, Delucchi, and Schneider (2008) [167], who reported a mean response rate of 44.4% with antidepressants compared to 34.7% with placebo, with a clearer effect emerging in longer studies. The data reviewed also indicate a higher rate of discontinuation due to adverse events among treated patients, underscoring the importance of careful clinical monitoring.

Among SSRIs, sertraline and escitalopram are regarded as first-line treatments. Sertraline is associated with a favorable safety profile and a low risk of pharmacokinetic interactions, a key consideration for patients who often take multiple medications concurrently [167,168]. Escitalopram, the enantiomer of citalopram, is likewise preferred for its good tolerability and lower likelihood of inducing gastrointestinal or cognitive side effects. Clinical data also demonstrate that escitalopram significantly reduces depressive symptoms in patients over 65 years, with a lower dropout rate compared with other SSRIs [167,169]. Citalopram has been widely used, but its use in older adults has become more cautious due to the dose-dependent risk of QT interval prolongation and potential arrhythmic complications, which limit recommended doses in the geriatric population [170].

Fluoxetine, by contrast, is generally avoided in the elderly because its long half-life and inhibition of the CYP2D6 enzyme substantially increase the risk of pharmacological interactions and drug accumulation, leading to adverse effects. The same caution applies to fluvoxamine, which should be avoided, particularly in patients on multiple medications, due to its high interaction potential [171].

SNRIs used in late-onset depression offer additional insights: Raskin et al. (2007) documented, in a sample with a mean age of 72 years, that duloxetine at 60 mg/day not only reduced depressive symptoms but also improved certain cognitive functions and chronic pain, with response and remission rates nearly twice those of placebo [166]. Venlafaxine, especially in its extended-release formulation and at doses above 150 mg/day, appears particularly suitable for cases marked by prominent apathy or psychomotor slowing, likely due to its more robust noradrenergic activity [169].

In terms of safety, several precautions remain essential. Hyponatremia, for example, is not uncommon, affecting approximately 9% of elderly patients treated with antidepressants [172]. Another critical concern is the increased risk of gastrointestinal bleeding when SSRIs are combined with NSAIDs [173,174]. Venlafaxine warrants specific mention for its dose-dependent potential to increase blood pressure, a consideration particularly relevant in patients with poorly controlled hypertension. Overall, escitalopram and sertraline are often preferred in more fragile or polytreated patients due to their minimal involvement in CYP450 metabolic pathways [175].

Multiple clinical guidelines confirm this orientation, recommending SSRIs and SNRIs as first-line agents for depression in late life, while emphasizing the need for vigilance regarding hyponatremia, falls, and QT prolongation. Ultimately, the optimal strategy is never “one drug fits all”: treatment selection must be personalized, taking into account comorbidities as well as potential risks.

#### 11.1.2. Pro-Dopaminergic and Stimulant Approaches

Late-onset depression tends to present with symptoms such as anhedonia, apathy, psychomotor retardation, and cognitive impairment—manifestations that are particularly relevant in the elderly population. In this context, antidepressants that directly modulate dopaminergic transmission have attracted increasing interest, as they target symptom domains that are often poorly addressed by SSRIs or SNRIs alone [61].

Bupropion is a norepinephrine–dopamine reuptake inhibitor (NDRI) and a non-competitive antagonist of nicotinic cholinergic receptors [176,177]. This synergistic action increases dopamine and norepinephrine availability in mesolimbic and cortical pathways, thereby improving motivation and counteracting symptoms of anergia. Several studies have evaluated its efficacy in older adults. In a 12-week naturalistic study of patients over 65 years with major depression, 67% achieved a clinical response and 50% reached partial or complete remission [178]. In a multicenter randomized trial of bupropion XL (150–300 mg/day), Hewett et al. (2010) reported significant reductions in MADRS scores compared with placebo, along with improvements in energy, motivation, and quality of life [179]. Its tolerability profile is also favorable: the most common adverse events are insomnia, dry mouth, and tremor [180], while the risk of seizures remains low at 0.4% for doses below 450 mg/day [181]. Compared with SSRIs, bupropion carries a lower risk of sexual dysfunction and weight gain, making it an optimal choice for patients with metabolic syndrome or concerns regarding quality of life [182]. For these reasons, bupropion represents one of the most suitable options for late-onset depression characterized by predominant apathy and anhedonia.

Pramipexole is a dopaminergic agonist with high affinity for D_2_/D_3_ receptors, approved for the treatment of Parkinson’s disease and restless legs syndrome. Over the past two decades, evidence has emerged supporting its antidepressant potential. A randomized trial conducted by Barone et al. (2010) [183] in 296 Parkinson’s patients showed that pramipexole significantly reduced BDI scores compared with placebo, independently of motor improvement—indicating a direct mood effect. In non-Parkinsonian populations, pramipexole has demonstrated efficacy as an augmentation therapy in treatment-resistant depression: in a 2025 British RCT, the addition of pramipexole to standard therapy tripled response rates (44% vs. 16%) and quadrupled remission rates (28% vs. 8%) after 12 weeks [184]. The benefits persisted up to 48 weeks, although 20% of participants discontinued due to adverse events (nausea, insomnia, dizziness). These data highlight the potential of pramipexole in treating anhedonia and apathy—symptom domains in which serotonin alone often proves ineffective. However, its tolerability requires caution, especially in frail elderly patients, in whom the risk of orthostatic hypotension and sleep disturbances should be carefully monitored.

Methylphenidate, a psychostimulant that inhibits dopamine and norepinephrine reuptake at the synaptic level, is traditionally used in attention-deficit/hyperactivity disorder (ADHD) [185]. In geriatric psychiatry, it has been investigated as an “accelerator” of antidepressant response and as an enhancer of conventional pharmacotherapy. A randomized, double-blind trial involving 143 older adults (mean age: 70 years) showed that the combination of citalopram + methylphenidate (5–40 mg/day) produced faster and greater clinical improvements than either drug alone [186]. By the fourth week, the combination group exhibited a significant reduction in depressive severity and higher remission rates, without an increase in adverse events compared to placebo. In an earlier study, Lavretsky and Kumar (2001) observed that methylphenidate augmentation led to rapid symptom improvement in elderly patients with a mean age above 80 years, with no treatment discontinuations [187]. A more recent systematic review and meta-analysis [188] confirmed that combining psychostimulants with antidepressants significantly reduces depressive symptoms, although it does not consistently increase remission rates. The use of methylphenidate therefore appears beneficial in cases where the therapeutic latency of conventional antidepressants represents a clinical challenge, though cardiovascular monitoring remains essential.

#### 11.1.3. Cognitive Enhancers

Late-onset depression is frequently associated with cognitive deficits, including impairments in memory, executive functioning, and attention. Unlike early-onset forms, late-onset depression more often co-occurs with cerebrovascular lesions, neuroinflammation, and reduced synaptic plasticity—all factors contributing to baseline cognitive decline and greater resistance to conventional treatments [189]. Moreover, physiological aging reduces acetylcholine levels and alters glutamatergic transmission, providing a clinical rationale for the use of pro-cognitive agents such as cholinesterase inhibitors and NMDA antagonists in elderly depressed patients [190,191]. Donepezil, rivastigmine, galantamine, and memantine have been investigated as potential cognitive enhancers in late-onset depression, given the frequent coexistence of mood and cognitive disturbances in the elderly.

Donepezil is a selective acetylcholinesterase inhibitor that increases acetylcholine availability in cortical and hippocampal regions. Its use in elderly depressed patients with cognitive deficits is based on the hypothesis that cholinergic enhancement may improve memory and executive functioning. In a randomized, placebo-controlled pilot study of patients over 50 years already receiving SSRIs, adjunctive donepezil (10 mg/day) produced significant improvements in episodic memory and executive function compared with placebo [190]. Subsequently, Reynolds et al. (2011) [191] evaluated donepezil in a long-term maintenance trial, observing cognitive benefits in patients with depression treated with antidepressants plus donepezil. Its safety profile includes mild side effects such as nausea and insomnia, while cardiovascular events like bradycardia and syncope are rare but warrant clinical monitoring.

Rivastigmine is an irreversible acetylcholinesterase inhibitor that acts both cortically and subcortically. It has been tested in complex clinical contexts such as treatment-resistant geriatric depression or depression associated with intensive neurobiological therapies. Van Schaik et al. (2015) reported in a case series that rivastigmine improved cognitive status in three elderly depressed patients who had developed cognitive deterioration following electroconvulsive therapy (ECT), particularly reducing confusion symptoms [192]. Similarly, the RECALL trial was designed to assess rivastigmine’s efficacy in reducing interictal delirium related to ECT. This multicenter, prospective study involves a cohort of 150 patients and a randomized placebo subgroup, aiming to clarify cognitive determinants linked to ECT and to provide preventive pharmacological options. These findings suggest that rivastigmine may play a specific role in managing cognitive complications related to somatic treatments in late-onset depression [193].

Galantamine acts as a reversible acetylcholinesterase inhibitor and an allosteric modulator of nicotinic receptors, exerting a dual effect on cholinergic enhancement. Holtzheimer et al. (2008) [194] conducted a randomized, placebo-controlled pilot study in 38 adults over 50 years with major depression but without dementia. Patients received galantamine adjunctive to antidepressants (venlafaxine or citalopram) or placebo for 24 weeks. Both groups showed significant improvements in mood and cognitive function, with no substantial differences between arms. However, a post hoc analysis indicated a faster reduction in depressive symptoms with galantamine during the first two weeks. Despite a high dropout rate, the study suggested that cholinergic enhancement might exert an early impact on depressive symptomatology.

Memantine, a low-affinity noncompetitive NMDA receptor antagonist, modulates glutamatergic excitotoxicity that may contribute to both cognitive deterioration and depression. Lenze et al. (2012) [195] conducted a randomized trial in 35 patients over 60 years hospitalized for rehabilitation after disabling medical events, presenting with depression (HAM-D ≥10) and/or apathy (AES ≥40). After 12 weeks, both groups (memantine and placebo) showed significant improvements in depressive symptoms and physical functioning, but no between-group differences or effects on apathy were found. In a subsequent study, Lavretsky et al. (2020) [196] evaluated the combination of escitalopram + memantine in 95 older adults with depression and subjective memory complaints. After 6–12 months, both groups showed mood improvements, but the ESC/MEM group demonstrated greater cognitive and neuropsychological benefits, suggesting a potential neurocognitive advantage without compromising antidepressant efficacy.

Taken together, these findings indicate that targeting cholinergic and glutamatergic pathways may offer promising adjunctive strategies for addressing the cognitive and motivational deficits frequently accompanying late-onset depression.

More recent approaches involve microRNA (miRNA), small interfering RNAs (siRNAs), piwi-associated small RNAs (piRNAs), and transfer RNA-derived small RNAs (tsRNAs) [197], treatments targeting oxidative stress [198,199], monoclonal anti-amyloid antibodies [200], DYRK1A inhibitors [201], and multi-target quinolines which address simultaneously cholinesterase activity, oxidative stress and amyloid aggregation [202].

#### 11.1.4. Management of Treatment-Resistant Late-Onset Depression (TR-LOD)

In late-life practice, treatment resistance usually means no meaningful clinical response after two adequate antidepressant trials in the current episode, verified for dose, duration, and adherence [156]. When first-line antidepressants fail in late life, switching to another antidepressant rather than combining agents is recommended. Where needed, augmentation with aripiprazole or lithium is suggested [203]. The choice among these paths should follow shared decision-making that weighs clinical status, prior treatment history, and patient preference [156]. Trials and guidelines typically define response as a ≥50% symptom reduction, but management in practice distinguishes no response, modest response, and significant improvement without remission, because next steps differ across these states. In no response, the recommendation is to switch (often across class) rather than augment an ineffective base; after two non-responses, the diagnosis should be re-examined (e.g., apathy, hypoactive delirium, drug-induced presentations). Before applying the label, reassess the symptom profile (including cognition), review drug–drug interactions and adverse-effect burden, and address medical or sleep comorbidities [153,204]. If depression is reconfirmed, neuromodulation—modified ECT or repetitive TMS (see below)—becomes the preferred option; a cautious trial of very low-dose sulpiride (30–50 mg/day) may be attempted before neuromodulation, acknowledging scant evidence and the risk of extrapyramidal symptoms [156]. A randomized, sham-controlled trial showed that TMS (left DLPFC 10 Hz, 120% MT, 4 weeks) improved immediate memory and attention without serious adverse events in LOD [205,206]. In modest response, either a switch (including within-class if there was some benefit) or augmentation with aripiprazole can be pursued [156,207]. For pharmacologic rescue, ketamine-class treatments offer rapid antidepressant effects in adult MDD/TRD with acceptable all-cause discontinuation rates; a 2023 meta-analysis (49 RCTs; n = 3299) found numerically greater efficacy for racemic ketamine than esketamine across acute, ongoing, and follow-up windows, with similar overall dropout to controls. The same synthesis catalogued elderly-focused trials (e.g., esketamine in older TRD in TRANSFORM-3; a pilot RCT of titrated subcutaneous ketamine in older TRD), supporting feasibility in late-life samples while highlighting that dedicated geriatric datasets remain relatively limited [208]. Beyond rescue strategies, pharmacogenetics can refine prescribing amid polypharmacy—CYP2C19/CYP2D6 status and SLC6A4/HTR2A/ABCB1 variants relate to tolerability and response, with combinatorial panels outperforming single-gene approaches [209]. The main pharmacological strategies for the treatment of late-onset depression are summarized in Table 2.

### 11.2. Neuromodulation Strategies

Given the limited efficacy and frequent adverse effects associated with pharmacological treatments in older adults—particularly in those with treatment-resistant depression—neuromodulation techniques have gained increasing prominence as both alternative and adjunctive strategies [210,211]. Among these, electroconvulsive therapy (ECT), repetitive transcranial magnetic stimulation (rTMS), and deep transcranial magnetic stimulation (dTMS) represent the most extensively studied and clinically utilized modalities, each with distinct therapeutic and neurophysiological profiles.

ECT remains the most effective intervention for severe or psychotic depression and continues to represent the treatment of choice in life-threatening or refractory cases [212]. However, its use in geriatric populations is often limited by the requirement for anesthesia [213] and by concerns regarding cognitive side effects [214], particularly in patients with comorbid neurocognitive disorders. These limitations have stimulated growing interest in non-invasive neuromodulatory alternatives, notably rTMS and dTMS, which offer safer and more easily applicable options for older adults.

rTMS has accumulated substantial evidence supporting its efficacy and safety in geriatric depression. A recent systematic review and meta-analysis including 10 randomized controlled trials evaluated the efficacy of repetitive transcranial magnetic stimulation (rTMS) in late-life depression. The pooled findings demonstrated a significant improvement in depressive symptomatology, as reflected by a reduction in Hamilton Depression Rating Scale (HDRS) scores, along with higher response and remission rates compared with sham stimulation [215].

Deep TMS (dTMS), which employs H-coils to stimulate deeper cortical and subcortical targets, may hold particular advantages in late-life depression, as age-related cortical atrophy increases the scalp–cortex distance, potentially limiting the efficacy of standard TMS coils. A large multicenter real-world study of 247 patients aged 60–91 years reported response and remission rates of 79.4% and 60.3%, respectively, after 30 dTMS sessions, with 78% achieving remission on clinician-rated HDRS-21 and no serious adverse events [216]. Consistent results were observed in a randomized controlled trial of 52 patients aged 60–85 years with major depression, where high-dose deep rTMS achieved 40.0% remission vs. 14.8% with sham, without cognitive decline or major adverse effects, aside from mild transient scalp pain [217].

Nevertheless, several limitations should be considered when interpreting these findings in older populations. Neuromodulation studies often involve relatively healthy and motivated patients [218], potentially introducing selection bias and limiting generalizability to frailer individuals with multiple medical comorbidities or advanced cognitive impairment. In addition, tolerability may vary in very old patients, and access to neuromodulation remains uneven due to availability of specialized centers, treatment costs, and logistical barriers. These factors underscore the need for careful patient selection and for further real-world studies specifically addressing feasibility and long-term outcomes in heterogeneous geriatric populations.

Emerging evidence supports rTMS and dTMS as safe, effective, and well-tolerated options for late-onset depression (LOD), particularly in cases of pharmacological non-response or intolerance. Their increasing integration into geriatric depression treatment algorithms underscores their value within a multimodal, patient-centered approach aimed at improving both mood and cognitive outcomes in older adults.

### 11.3. Psychotherapy and Other Non Pharmacological Intervention

Psychotherapy is a well-established form of treatment for depression, featuring in all major guidelines [219,220,221]. Several manualized, protocol-based psychotherapies have been validated as effective treatments for depression, though some possess more robust empirical support than others. Cognitive Behavioral Therapy (CBT), Interpersonal Therapy (IPT), and Behavioral Activation (BA) are all considered first-line interventions for mild to moderate depressive episodes and are recommended as adjunctive treatments to pharmacotherapy in more severe cases. The following section discusses how these evidence-based approaches can be adapted to address the specific clinical and neurocognitive features of late-onset depression.

#### 11.3.1. Cognitive Behavioral Therapy

CBT is the most extensively studied form of psychotherapy and is often regarded as the gold standard among psychological treatments for a wide range of mental disorders [222]. Its theoretical foundations lie in Stoic philosophy and constructivism, emphasizing that the interpretation of reality plays a central role in the development and maintenance of psychiatric disorders. CBT also integrates key principles of behaviorism, particularly the concept that exposure to feared stimuli is essential for reducing maladaptive arousal responses [222,223]. Aaron T. Beck, considered as the father of Cognitive Therapy, introduced the concept of the “cognitive triad” of depression—negative thoughts about the self, the world, and the future [224]—which arise from cognitive distortions such as catastrophizing, arbitrary inference, and minimization. CBT for depression aims to identify and challenge these distortions through cognitive restructuring, thereby fostering more adaptive patterns of thinking, while simultaneously encouraging behavioral activation and the reinforcement of healthy behaviors. CBT is still under-investigated as a psychotherapeutic approach for LOD and, more broadly, LLD. Although recent randomized controlled evidence, such as the trial by Dafsari et al. (2023) [225], found no superiority of LLD-specific CBT over supportive treatment, both interventions produced meaningful clinical benefits in patients with moderate-to-severe LLD. The methodological rigor of these studies nevertheless highlights the feasibility and potential value of structured psychotherapies in this population, supporting their inclusion within multimodal treatment frameworks for late-life mood disorders. Combination therapies involving CBT and pharmacotherapy may be more testable for both LLD and LOD, as placebo psychotherapies are unlikely to be rendered double-blinded.

However, conclusions can be drawn from research on the broader category of late-life depression (LLD), which includes elderly patients with both early- and late-onset forms of the disorder. Several randomized trials and studies have demonstrated the effectiveness of CBT as a first-line treatment for LLD [225,226,227]. Nonetheless, treatment protocols must be adapted to the specific characteristics of this population. It is crucial to evaluate the feasibility of behavioral components and to tailor the complexity of cognitive restructuring and Socratic dialogue when patients present with cognitive impairments. Moreover, in LOD—distinct from LLD with early-onset depression—the therapeutic focus should often center on the life event that precipitated the depressive episode (e.g., bereavement, retirement, loss of autonomy), exploring how the patient’s interpretation of that event contributes to excessive emotional distress and impedes the development of resilient coping mechanisms.

#### 11.3.2. Behavioral Activation

Since its original introduction into psychiatry, CBT has undergone significant evolution, giving rise to what is now referred to as the “third wave” of CBT—an approach that integrates mindfulness, meditation, and acceptance-based techniques alongside traditional cognitive and behavioral strategies. However, empirical studies and expert consensus consistently emphasize that the behavioral component remains the pivotal element driving therapeutic efficacy [228,229]. This emphasis is reflected in modern clinical guidelines, which increasingly recommend specific behavioral therapies rather than broadly prescribing generic CBT, thereby underscoring the importance of structured, protocol-based interventions tailored to well-defined behavioral targets [219,230].

Building upon the behavioral foundations emphasized within CBT, Behavioral Activation (BA) represents a focused, protocol-driven approach that operationalizes these principles into a structured therapeutic framework. BA aims to help patients re-engage in pleasant and meaningful activities, particularly when intrinsic motivation is diminished, thereby encouraging them to act in order to feel better, rather than waiting to feel better before acting. This approach directly disrupts one of the central maintaining mechanisms of depression—the cycle of passivity and withdrawal [231,232,233]. BA has demonstrated strong efficacy in the treatment of depression, showing large effect sizes and possibly representing the most effective standalone psychological intervention available [234]. While no studies have specifically investigated its use in LOD, substantial evidence supports its effectiveness in LLD [235,236]. However, protocol adaptations may be necessary to accommodate the functional and medical comorbidities common among older adults [237]. In the specific context of LOD, it remains essential to identify and address life stressors—such as bereavement, retirement, or significant changes in routine—that may have triggered the depressive episode, thereby tailoring the intervention toward restoring behavioral engagement and adaptive daily structure following such life-altering events.

#### 11.3.3. Interpersonal Therapy

Interpersonal Therapy (IPT) is a structured, attachment-focused psychotherapy that conceptualizes psychological distress as arising from difficulties in forming and maintaining satisfying interpersonal relationships. The treatment focuses on key interpersonal themes such as unresolved grief, role transitions, and interpersonal conflicts, aiming to improve social functioning and reduce depressive symptoms [238,239]. IPT has been integrated into major clinical guidelines for the treatment of depression, supported by robust evidence for its efficacy across various populations [240]. As with the previously discussed psychotherapies, no studies have specifically examined the effectiveness of IPT in late-onset depression (LOD); however, there is substantial evidence demonstrating its benefits in late-life depression (LLD), with only minimal protocol modifications required for older adults [241]. This adaptability is largely attributable to the fact that many of the precipitating factors commonly seen in both LOD and LLD—such as bereavement, retirement, role transitions, and significant relational changes within the household or workplace—are inherently central to the IPT framework, making it a particularly suitable and conceptually coherent intervention for this population.

In Table 3, we summarize the main evidence-based psychotherapeutic approaches for LOD, highlighting their core principles and the specific adjustments required to address the clinical, functional, and cognitive characteristics commonly observed in older adults.

#### 11.3.4. Lifestyle Interventions

There is growing evidence that lifestyle factors, particularly diet and physical activity, play a crucial role in the onset, course, and treatment response of LOD [242]. Nutritional patterns rich in omega-3 fatty acids, olive oil, fruits, vegetables, and brain-supportive macro- and micronutrients have consistently been associated with a lower risk of depressive symptoms in older adults, acting as protective factors that sustain neuronal integrity and reduce systemic inflammation [243].

Equally important is the role of physical exercise, which exerts multifaceted antidepressant effects through neurobiological, cognitive, and psychosocial mechanisms. Exercise promotes neurogenesis, improves cerebral perfusion, modulates monoaminergic systems, and enhances endorphin release—all of which contribute to mood regulation and cognitive resilience [244,245,246]. A study comparing patients treated with sertraline plus structured physical exercise versus sertraline alone found that those in the combined intervention group achieved remission more rapidly [247], underscoring the additive effect of movement-based interventions.

In the elderly, exercise also carries unique psychosocial benefits. Participating in group-based physical or recreational activities not only reinforces routine and behavioral activation but also mitigates loneliness and social isolation, both of which are potent risk factors and maintaining mechanisms for LOD [248,249,250,251]. Regular engagement in socially stimulating environments—such as walking groups, community classes, or light sport activities—provides continuous positive feedback, helping patients experience a sense of achievement and mastery that counteracts apathy and anhedonia.

At the same time, addressing maladaptive lifestyle habits, such as smoking and excessive alcohol consumption, is essential, as these behaviors exert disproportionately harmful effects in older adults compared with younger populations [252,253].

### 11.4. Therapeutic Considerations and Integrative Treatment Framework

Taken together, these findings highlight that integrative treatment approaches in late-onset depression should not be conceptualized as uniform or additive by default. Pharmacological and neuromodulatory interventions primarily target core affective symptoms and underlying biological vulnerability, whereas psychotherapeutic approaches address coping strategies, illness representation, and treatment adherence, and lifestyle interventions mainly modulate vascular risk, physical functioning, and social engagement. The relative contribution and sequencing of these interventions may vary according to individual clinical profiles, including cognitive status, medical comorbidity, psychosocial context, and stage of disorder. Accordingly, effective management of late-onset depression requires personalized, multimodal strategies rather than standardized treatment packages.

## 12. Conclusions, Future Perspective and Viewpoints

LOD sits at a difficult crossroad where medicine meets biography. It is challenging for clinicians—because treatment must be tailored to ageing bodies, multiple morbidity, and polypharmacy—and it is challenging for patients, who confront a first MDE after a lifetime of experience, roles, and meanings already settled. The existential dimension deserves equal weight: losses, loneliness, and the reappraisal of one’s life story often shape symptom expression and therapeutic response as much as neurobiology does.

Although relatively uncommon, LOD warrants special attention. It is not simply “depression happening in older people.” It is not merely depression occurring in older age, but rather a form of depressive illness shaped by aging-related biological, cognitive, and psychosocial processes. Differently from LLD, LOD does not involve a loss of the ability to adapt to stress, but is rather the consequence of vascular changes [254].

It differs from EOD and only partially overlaps with LLD driven by recurrent courses. Seeing LOD from the patient’s vantage point is essential: a first-ever episode in late life has a different symptomatology, prognosis, and set of needs than recurrent illness resurfacing in old age. Empathy helps, but so must epistemic humility—an effort to understand the world around an older adult whose convictions may be more firmly held, whose resources and vulnerabilities are uniquely configured.

Looking ahead, prevention is as important as treatment. As longevity increases, we should expect more LOD, not less. Public health measures that reduce loneliness and social isolation—age-friendly communities, accessible cultural and intergenerational spaces, proactive primary-care screening, and social prescribing—are likely to yield dividends. Clinical services should integrate psychiatry, geriatrics, neurology, and rehabilitation, with systematic cognitive monitoring and attention to vascular and metabolic health.

Scientifically, LOD offers a fertile test bed for bridging psychodynamic psychiatry and neuroscience: life-story dynamics, attachment, and meaning-making operate alongside cerebrovascular change, neuroinflammation, and altered plasticity. The field now needs longitudinal cohorts and pragmatic intervention trials that combine biological targets (vascular risk reduction, anti-inflammatory and pro-plasticity strategies, dopaminergic approaches when apathy and anergia dominate) with psychologically informed therapies adapted to late life. Recognizing LOD as a distinct clinical trajectory, rather than a discrete disorder, may help refine risk stratification, diagnostic reasoning, and personalized treatment planning in later life. Multimodal, personalized care—measuring what matters to older adults and iteratively adjusting treatment—should be our organizing principle.

## Figures and Tables

**Table 1:** Demographic, psychological, and environmental factors associated with increased risk for Late-Onset Depression (LOD).

Category	Risk Factor	Description
**Demographic factors**	Advanced age Female gender Low educational level Living alone/widowhood Low socioeconomic status	Increased prevalence with age, particularly aged between 70 and 80 Higher incidence in women, possibly related to hormonal and social factors Associated with lower health literacy and cognitive reserve Linked to social isolation and lack of emotional support Financial stress, limited access to care
**Psychological factors**	Social isolation Loss of spouse, friends, or family Lack of social support Institutionalization or nursing home residence History of trauma or abuse Recent stressful life events (e.g., illness, relocation)	Major contributor to emotional distress in older adults Bereavement is a common trigger of depressive episodes Increases vulnerability to depression Often associated with feelings of abandonment, helplessness Especially relevant in those with unresolved early-life adversity Psychological stressors may precipitate depressive symptoms
**Cultural and Environmental factors**	Stigma toward mental illness Limited access to mental health services	Delays in diagnosis and access to treatment Particularly in rural or underserved areas

**Table 2:** Summary of pharmacological strategies for the treatment of Late-Onset Depression (LOD). The table outlines the main therapeutic classes, representative agents, mechanisms of action, and key clinical considerations in older adults. Data emphasize the balance between efficacy and tolerability, the importance of targeting core features such as apathy and cognitive decline, and the need for careful pharmacovigilance in polytreated or frail patients.

Drug Class	Agents/Mechanism of Action	Clinical Features/Main Findings	Advantages	Limitations/Safety Concerns
**SSRIs and SNRIs**	SSRIs (sertraline, escitalopram, citalopram, fluoxetine, fluvoxamine); SNRIs (venlafaxine, duloxetine)	First-line options for LOD; act on serotonergic (± noradrenergic) transmission to modulate fronto-limbic circuits. SNRIs beneficial for apathy, psychomotor retardation, and pain.	Broad efficacy, established safety data, cognitive and somatic symptom improvement.	Hyponatremia (~9%), QT prolongation (citalopram), bleeding with NSAIDs, BP elevation (venlafaxine), CYP interactions (fluoxetine, fluvoxamine).
**Pro-dopaminergic and** **Stimulant Approaches**	Bupropion (NDRI), Pramipexole (D_2_/D_3_ agonist), Methylphenidate (DA/NE reuptake inhibitor)	Target anhedonia, apathy, psychomotor retardation, and cognitive slowing—symptoms less responsive to SSRIs/SNRIs.	Improve motivation, energy, and mood; lower risk of sexual dysfunction (bupropion); rapid onset (methylphenidate).	Insomnia, dry mouth, tremor (bupropion); orthostatic hypotension and sleep disturbances (pramipexole); cardiovascular monitoring required (methylphenidate).
**Cognitive Enhancers**	Cholinesterase inhibitors (donepezil, rivastigmine, galantamine); NMDA antagonist (memantine)	Address cognitive and executive deficits common in LOD; adjunctive use with antidepressants.	Potential cognitive and functional improvement; suitable for depression with cognitive impairment.	Nausea, insomnia, bradycardia (donepezil); confusion reduction post-ECT (rivastigmine); limited efficacy data (memantine).
**Management of Treatment-Resistant LOD**	Strategy-based: switch, augment (aripiprazole, lithium), neuromodulation (ECT, rTMS), ketamine/esketamine, pharmacogenetics	No response after ≥2 adequate antidepressant trials = TR-LOD. Switching preferred before augmentation; neuromodulation for resistant cases. Ketamine-class agents emerging; pharmacogenetics for precision prescribing.	Structured algorithms, evidence-based escalation, potential rapid effects (ketamine), integration with shared decision-making.	Polypharmacy risks, cognitive comorbidity, need for BP/cardiac monitoring (ketamine, TMS), limited geriatric RCTs.

Notes: BP, Blood Pressure; CYP, Cytochrome P450; DA, Dopamine; ECT, Electroconvulsive Therapy; LOD, Late-Onset Depression; NE, Norepinephrine; NDRI, Norepinephrine–Dopamine Reuptake Inhibitor; NMDA, N-methyl-D-aspartate; RCT, Randomized Controlled Trial; rTMS, repetitive Transcranial Magnetic Stimulation; SNRI, Serotonin–Norepinephrine Reuptake Inhibitor; SSRI, Selective Serotonin Reuptake Inhibitor; TR-LOD, Treatment-Resistant Late-Onset Depression.

**Table 3:** Summary of the main evidence-based psychotherapeutic approaches for late-onset depression (LOD), outlining their core principles and the specific adjustments required to accommodate the clinical and cognitive characteristics of older adults. Notes: BA, Behavioral Activation; CBT, Cognitive Behavioral Therapy; IT, Interpersonal Therapy; LOD, Late-Onset Depression.

Psychotherapy	Principles	Adjustments for LOD
CBT	Identifying cognitive distortions in the interpretation of reality and fostering more adaptive patterns of thinking and behavior.	Tailoring cognitive restructuring and the overall complexity of the intervention to the patient’s individual needs and abilities.
BA	Engaging in pleasant and meaningful activities to disrupt the vicious cycle of withdrawal and inactivity.	Verifying the feasibility of proposed activities while considering the patient’s potential physical or cognitive limitations.
IT	Identifying interpersonal triggers contributing to depression and developing effective strategies to improve these situations.	There are virtually no adjustments required.

## Data Availability

Not applicable. No new data were generated.

## Abbreviations

The following abbreviations are used in this manuscript:

ABCB1	ATP-Binding Cassette Subfamily B Member 1
AD	Alzheimer’s Disease
ADHD	Attention-Deficit/Hyperactivity Disorder
AES	Apathy Evaluation Scale
APA	American Psychological Association
BA	Behavioral Activation
BBB	Blood–Brain Barrier
BDI	Beck Depression Inventory
BDNF	Brain-Derived Neurotrophic Factor
BMI	Body Mass Index
CBT	Cognitive Behavioral Therapy
CESD-10	Center for Epidemiologic Studies Depression Scale (10-item version)
CNS	Central Nervous System
CRP	C-Reactive Protein
CSF	Cerebrospinal Fluid
CSVD	Cerebral Small Vessel Disease
CYP2C19	Cytochrome P450 2C19
CYP2D6	Cytochrome P450 2D6
CYP450	Cytochrome P450
DAMPs	Damage-Associated Molecular Patterns
DLPFC	Dorsolateral Prefrontal Cortex
DSM-5	*Diagnostic and Statistical Manual of Mental Disorders, Fifth Edition*
dTMS	Deep Transcranial Magnetic Stimulation
ECT	Electroconvulsive Therapy
EOD	Early-Onset Depression
ESC/MEM	Escitalopram + Memantine
FDA	U.S. Food and Drug Administration
FTD	Frontotemporal Dementia
GDS	Geriatric Depression Scale
HAM-D	Hamilton Depression Rating Scale
HDRS	Hamilton Depression Rating Scale
HDRS-21	21-item Hamilton Depression Rating Scale
HPA	Hypothalamic–Pituitary–Adrenal (axis)
HTR2A	5-Hydroxytryptamine (Serotonin) Receptor 2A Gene
IADL	Instrumental Activities of Daily Living
IL-6	Interleukin-6
IPT	Interpersonal Therapy
LLD	Late-Life Depression
LOD	Late-Onset Depression
MADRS	Montgomery–Åsberg Depression Rating Scale
MDD	Major Depressive Disorder
MDE	Major Depressive Episode
MEM	Memantine
MRI	Magnetic Resonance Imaging
MT	Motor Threshold
NDRI	Norepinephrine–Dopamine Reuptake Inhibitor
NMDA	N-Methyl-D-Aspartate
NLRP3	NOD-, LRR-, and Pyrin Domain-Containing Protein 3
NSAIDs	Nonsteroidal Anti-Inflammatory Drugs
OMEGA-3	Omega-3 Polyunsaturated Fatty Acids
PET	Positron Emission Tomography
PROSPECT	Prevention of Suicide in Primary Care Elderly: Collaborative Trial
RCT	Randomized Controlled Trial
ROS	Reactive Oxygen Species
rTMS	Repetitive Transcranial Magnetic Stimulation
SLC6A4	Solute Carrier Family 6 Member 4 (Serotonin Transporter Gene)
SNRI	Serotonin–Norepinephrine Reuptake Inhibitor
SSRI	Selective Serotonin Reuptake Inhibitor
TMS	Transcranial Magnetic Stimulation
TNF-α	Tumor Necrosis Factor-Alpha
TRD	Treatment-Resistant Depression
TR-LOD	Treatment-Resistant Late-Onset Depression
WMH	White Matter Hyperintensities
